# Separable structural requirements for cDNA synthesis, nontemplated extension, and template jumping by a non-LTR retroelement reverse transcriptase

**DOI:** 10.1016/j.jbc.2022.101624

**Published:** 2022-01-21

**Authors:** Sydney C. Pimentel, Heather E. Upton, Kathleen Collins

**Affiliations:** Department of Molecular and Cell Biology, University of California at Berkeley, Berkeley, California, USA

**Keywords:** reverse transcription, nucleic acid enzymology, biotechnology, RNA, silkworm, polymerase, non-LTR retroelement, nucleotide addition, terminal transferase, cDNA, complementary DNA, EN, endonuclease, LINE, long interspersed nuclear element, LTR, long terminal repeat, NTA, nontemplated nucleotide addition, RdRP, RNA-dependent RNA polymerase, RT, reverse transcriptase, TPRT, target-primed reverse transcription

## Abstract

Broad evolutionary expansion of polymerase families has enabled specialization of their activities for distinct cellular roles. In addition to template-complementary synthesis, many polymerases extend their duplex products by nontemplated nucleotide addition (NTA). This activity is exploited for laboratory strategies of cloning and sequencing nucleic acids and could have important biological function, although the latter has been challenging to test without separation-of-function mutations. Several retroelement and retroviral reverse transcriptases (RTs) support NTA and also template jumping, by which the RT performs continuous complementary DNA (cDNA) synthesis using physically separate templates. Previous studies that aimed to dissect the relationship between NTA and template jumping leave open questions about structural requirements for each activity and their interdependence. Here, we characterize the structural requirements for cDNA synthesis, NTA, template jumping, and the unique terminal transferase activity of *Bombyx mori* R2 non-long terminal repeat retroelement RT. With sequence alignments and structure modeling to guide mutagenesis, we generated enzyme variants across motifs generally conserved or specific to RT subgroups. Enzyme variants had diverse NTA profiles not correlated with other changes in cDNA synthesis activity or template jumping. Using these enzyme variants and panels of activity assay conditions, we show that template jumping requires NTA. However, template jumping by NTA-deficient enzymes can be rescued using primer duplex with a specific length of 3′ overhang. Our findings clarify the relationship between NTA and template jumping as well as additional activities of non-long terminal repeat RTs, with implications for the specialization of RT biological functions and laboratory applications.

Reverse transcriptases (RTs) are an evolutionarily diverse group of enzymes defined by their ability to synthesize DNA from an RNA template. Reverse transcriptase activity is fundamental to the perpetuation of selfish retroelements in all domains of life (1). In addition, RTs function in bacterial host defense, generate sequence diversity in phage and bacterial genes, and maintain eukaryotic chromosome ends (1, 2). From a biotechnological perspective, their ability to synthesize complementary DNA (cDNA) from both RNA and DNA templates is routinely exploited for laboratory and clinical applications including RNA-seq and RT-PCR. However, despite remarkable RT phylogenetic diversity, most applications rely exclusively on the recently branched retroviral RTs engineered by iterations of mutagenesis over the past several decades (3). Other retroelements include the eukaryotic long terminal repeat (LTR) retroelements, from which retroviral RTs emerged, and also the more ancestral non-LTR retroelements (4, 5). Unlike their evolutionary predecessors, retroviral RT active site features are adapted to benefit virus reproduction rather than perpetuation within the host genome. Retroviral enzymes are therefore insufficient model systems for understanding the complexity of selfish retroelement RT structures and activities. Understanding the properties of these retroelement RTs will provide a foundation for further optimization of biotechnological applications as well as insights into the RT properties necessary for retroelement propagation.

Sequence alignments and structural characterization support an overall RT active site architecture that can be envisioned as a right-handed arrangement of fingers, palm, and thumb subdomains (6, 7). Reverse transcriptases and also viral RNA-dependent RNA polymerases (RdRPs) share seven primary sequence motifs, 1 to 7 (8, 9). RNA-dependent RNA polymerases and non-viral RTs also share subsets of additional insertion motifs (Fig. 1*A*), typically termed 2a, 3a, 4a, 6a, and 7a, as well as an N-terminal extension that can include motifs 0 and −1 (10, 11, 12). Structure determination and mutagenesis studies of a limited number of bacterial mobile group II intron RTs and eukaryotic telomerase RTs indicate that motif insertions are tailored by evolution to adapt RTs to their biological functions (13, 14, 15, 16, 17).

**Figure 1:**
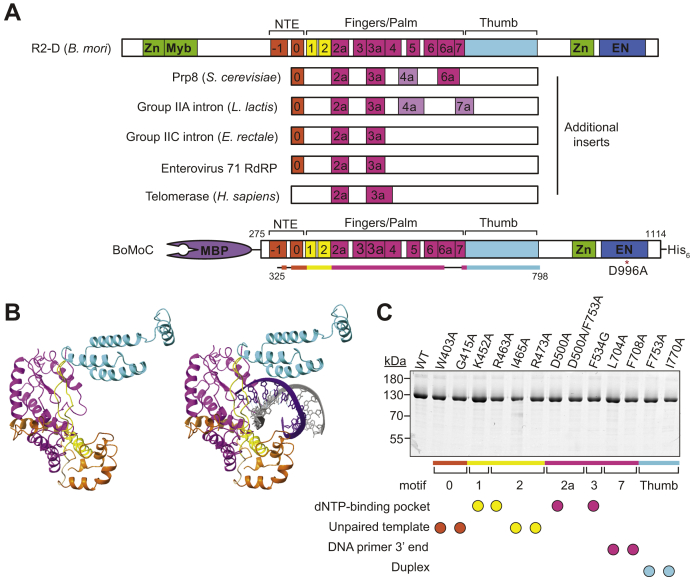
**Domain organization and structure prediction of BoMoC.***A*, *top*, schematic of the full-length *B. mori* R2 D-clade element including N-terminal zinc finger (Zn) and Myb domains and C-terminal Zn and EN domains. Other domains are described in the main text. Reverse transcriptase domain inserts beyond the conserved motifs 1 to 7 and thumb are shown as present in *B. mori* R2 protein and related proteins: Prp8 (*Saccharomyces cerevisiae*), Group IIA intron RT (*Lactococcus lactis*), Group IIC intron RT (*E. rectale*), Enterovirus 71 RdRP, and telomerase (*Homo sapiens*). Inserts not present in *B. mori* R2 protein are colored *light purple*. *Bottom*, schematic of BoMoC with N-terminal MBP tag and a C-terminal 6x-Histidine tag included. *Red asterisk* indicates the EN-inactivating D996A substitution. The *colored line* below represents amino acids modeled by Phyre2, and *thin black line* indicates residues modeled *ab initio* and not included in the structure renderings shown. *B*, BoMoC structure prediction shown without or including an RNA template (*gray*)-DNA primer (*purple*) duplex placed using the structure of thermostable group II intron RT (PDB 6AR3). *C*, sodium dodecyl sulfate - polyacrylamide gel electrophoresis gel of the purified BoMoC sequence variants analyzed in this study. Below the gel is a motif annotation color-coded as in (*A*) and used for all of the Figures. *Colored circles* indicate type of nucleotide or nucleic acid contacts that each residue is predicted to make. EN, endonuclease; MBP, maltose-binding protein; RdRP, RNA-dependent RNA polymerase; RT, reverse transcriptase.

Compared to retroviral, intron, and telomerase RTs, relatively little is known about eukaryotic non-LTR retroelement RT structure-function relationships. Non-LTR retroelement subgroups are distinguished by the number of ORFs and the presence of additional domains in the ORF encoding the RT (4, 18). A widespread early-branching group has a single ORF, with the central RT domain followed by a C-terminal endonuclease (EN) domain with a restriction enzyme-like EN fold. Members of this group often insert site-specifically, enabled by N-terminal sequence-specific DNA-binding domains (19). Site-specific insertion at a repetitive locus safeguards the host genome, because a few target-site disruptions can occur without compromise to organism fitness (4). A later-branching non-LTR retroelement group is distinguished by its typical encoding of two ORFs: an ORF1 RNA-binding protein that in in some retroelement lineages has other roles as well and an ORF2 protein with an N-terminal apurinic/apyrimidinic EN domain followed by an RT domain (4, 5). This group is generally less specific for an insertion site. For example, the human long interspersed nuclear element 1 (LINE-1) two-ORF retroelement is selective for only about 6 bp of target site sequence. Consequently, it has proliferated to constitute about a fifth of the human genome, and its ongoing mobility is implicated in oncogenesis (5).

Insertion of non-LTR retroelements occurs by concerted action of their RT and EN domains. The target-primed reverse transcription (TPRT) mechanism was first shown for the site-specific R2 retroelement from the silkmoth, *Bombyx mori* (20) and later for human LINE-1 (21). In TPRT, a target-site nick created by the EN domain is used to initiate cDNA synthesis on the RT-bound RNA template. Very little or no base-pairing of primer and template is required, notably different from the more extensive base-pairing required for initiation by LTR retroelement or retroviral RTs (3). Use of a template that has little or no ability to base-pair with a primer 3′ end is described as template jumping (22, 23), distinct from the template switching of retroviral RTs mediated by annealing of a cDNA liberated by RNase H to a complementary sequence in another template molecule (24).

Although TPRT is an activity specific to non-LTR retroelement RTs, other RNA-templated polymerases including RdRPs, group II intron RTs, and retroviral RTs show some ability to template jump under individually optimized reaction conditions *in vitro* (25, 26, 27, 28, 29). Template-jumping polymerases share the ability to extend their cDNA duplexes by additional nontemplated nucleotide addition (NTA), but the extent and nucleotide specificity of NTA vary dramatically, as do conclusions about the role of NTA in enabling or inhibiting template jumping (23, 27, 30, 31, 32, 33, 34, 35, 36, 37, 38).

We sought to determine the structural features of a eukaryotic non-LTR retroelement RT that allow it to perform NTA and template jumping and to investigate the interrelation of these two activities. We designed a library of over 100 sequence variants of *B. mori* R2 RT with side-chain substitutions across the N-terminal extension, fingers, palm, and thumb subdomains. Mutations were guided by multiple sequence alignment and protein structure prediction. Overall, our results provide the most detailed analysis to date of the structural requirements for the activities of a non-LTR retroelement RT. Among our conclusions, we demonstrate that NTA is a prerequisite for template jumping but can be bypassed using a primer duplex with the optimal length of 3′ overhang. Our findings provide insight into the requirements for cDNA synthesis initiation by the TPRT mechanism of non-LTR retroelement mobility and will enable expansion of RT applications to research and medicine.

## Results

### Design and cDNA synthesis activity of RT sequence variants

As a model non-LTR retroelement RT, we used a highly active, extensively purified version of *B. mori* R2 protein, designated BoMoC, which lacks the N-terminal sequence-specific DNA-binding domains (Fig. 1*A*, bottom) (29). To guide structure/function analysis, we used multiple sequence alignments to define the boundaries of shared RT motifs in BoMoC. The resulting sequence annotations largely agree with those reported by the Eickbush and Christensen labs (10, 12), with minor adjustments (Fig. S1). In addition, we generated a protein structure prediction for the R2 RT domain using Phyre2 (Fig. 1*B*). Both the calciviral rabbit hemorrhagic RdRP (PDB 1KHV) and a thermostable group II intron RT (PDB 6AR3) were used as templates to create the R2 RT structure prediction (7, 15). Additional comparisons used the structure of the spliceosome protein Prp8 (39), which contains an inactivated RT domain speculated to be present due to evolution of the spliceosome from a mobile element ribonucleoprotein.

Mutagenesis prioritization used alignments and structure modeling to predict residues important for the specificity and efficiency of dNTP incorporation by templated and nontemplated addition as well as residues potentially involved in binding primer and template nucleic acids. Ultimately, we selected side chains in motifs 0, 1, 2, 2a, 3, 7, and the thumb domain for substitution. Below, we describe the sequence-variant RTs with informative changes in product synthesis profile. BoMoC amino acid substitutions are numbered with respect to the ORF initially reported by the Eickbush lab (20), for comparison to full-length R2 RT. All side chain substitutions were to alanine or glycine. Enzyme variants were purified with Nickel-NTA resin followed by size-exclusion chromatography. Protein purity was confirmed by denaturing sodium dodecyl sulfate - polyacrylamide gel electrophoresis and direct protein staining (Fig. 1*C*).

**Figure 2:**
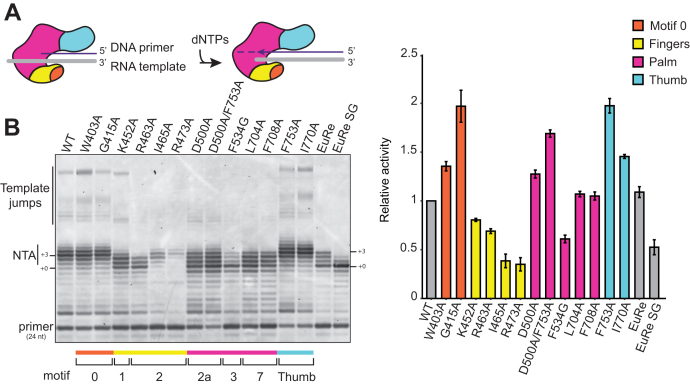
**BoMoC variants with altered NTA activity.***A*, diagram of BoMoC engaged with RNA template (*gray*)-DNA primer (*purple*) duplex for fill-in cDNA synthesis. *Dashed purple line* indicates NTA. *B*, *left*, SYBR-gold stained denaturing urea-PAGE gel of BoMoC and EuRe cDNA extension products. Labels indicate products with +0 and +3 NTA. *Right*, quantification of cDNA products from fill-in synthesis, not including products labeled Template Jumps, normalized to WT. cDNA, complementary DNA; NTA, nontemplated nucleotide addition.

BoMoC variants were first screened for the ability to synthesize cDNA on an annealed primer-template substrate, using a DNA primer that is 10 nt shorter than the RNA template (Fig. 2*A*; oligonucleotide sequences are listed in Table 1). Enzymes that were inactive or indistinguishable from the WT BoMoC in their product profile were dropped from subsequent assays. Most substitutions maintained or reduced enzyme activity, but substitutions in motif 0, motif 2a, and the thumb domain increased product synthesis relative to WT BoMoC (Fig. 2*B*).

**Table 1:** Oligonucleotide sequences

Figure	Type	Oligonucleotide	Sequence
2	RNA	Primer extension template	AGAUCGGAAGAGCACACGUCUGAACUCCAGUCAC/3SpC3
	DNA	Primer 1 (-10 fill in)	GTGACTGGAGTTCAGACGTGTGCT
4	RNA	Primer extension Cy3 template	/5Cy3/ATTCAACCCCAAAAATCTAGTGCTG
	DNA	Primer 2 (-8 fill in)	CAGCACTAGATTTTTGG
5,6	RNA	M13 (-) RNA	UCAUAGCUGUUUCCUGUGUGA
6	DNA	Universal DNA primer	GTGACTGGAGTTCAGACGTGTGCTCTTCCGATC
	DNA	Universal DNA primer +1T	GTGACTGGAGTTCAGACGTGTGCTCTTCCGATCT
	DNA	Universal DNA primer +2TC	GTGACTGGAGTTCAGACGTGTGCTCTTCCGATCTC
	DNA	Universal DNA primer +3TCA	GTGACTGGAGTTCAGACGTGTGCTCTTCCGATCTCA
	RNA	Universal primer complement	GAUCGGAAGAmGmCmAmCmAmCmGmUmCmUmGmAmAmCmUmCmCmAmGmU/3SpC3

Ribose substitutions with 2′ O-methyl ribose are indicated as “m” before the base. “3SpC3” indicates a three-carbon blocking group to prevent use of the oligonucleotide as a primer for DNA synthesis, and “5Cy3” is a 5′ Cy3 dye added to inhibit use of the oligonucleotide as a jump-acceptor template. For the oligonucleotides used in Figures 2 and 4, the primer was preannealed to the primer extension template. Figure 6 primer oligonucleotides were each preannealed to the primer complement. M13 (-) RNA was used as primer in terminal transferase assays (Fig. 5) or as a jump template (Fig. 6).

### BoMoC variants with altered NTA

Upon completion of template copying, full-length *B. mori* R2 protein extends its cDNA product by up to three additional non-templated nt (23). Using BoMoC, NTA can be detected up to 7 nt beyond the end of the template, with strong +3 and +4 NTA products (29). BoMoC finger and palm substitutions heterogeneously decreased NTA without parallel influence on the level of cDNA synthesis activity overall (Fig. 2*B*). Among the BoMoC enzymes compromised for NTA, BoMoC K452A, R463A, I465A, R473A, and F534G had decreased relative activity whereas BoMoC D500A, L704A, and F708A had increased relative activity compared to WT BoMoC (Fig. 2*B*).

**Figure 3:**
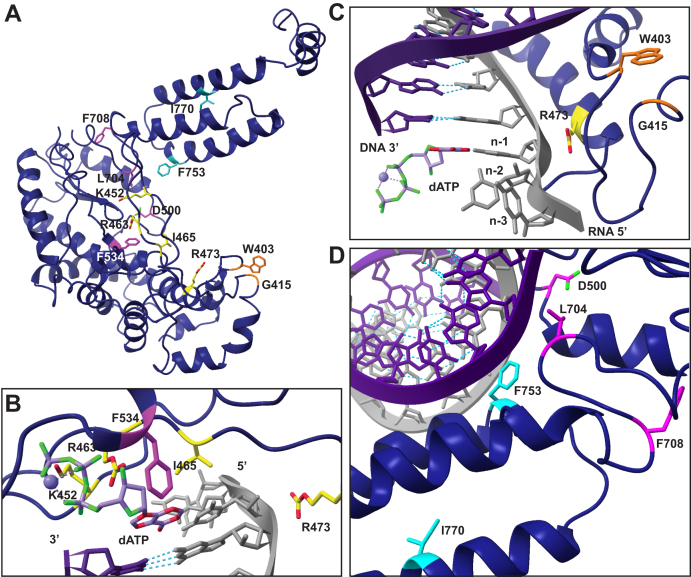
**Side chains targeted for investigation.***A*, BoMoC ribbon model with colored side chains shown for the residues substituted in work described here. All side chains that were examined in this study are labeled. *B* and *C*, close-up view of dNTP-binding pocket and motif 0 residues and their proximity to unpaired RNA template and the incoming nt (dATP). RNA template (*gray*)-DNA primer (*purple*) duplex and the incoming dATP were placed using the structure of thermostable group II intron RT (PDB 6AR3). *Light purple sphere* indicates Mg^2+^ ion coordinated to the beta and gamma phosphates. Oxygen atoms are colored *green* and nitrogen atoms are colored *red*. Only relevant secondary structure is shown for simplicity. Unpaired template residues are labeled, with the n-1 template nt positioned to base-pair with incoming nt. *D*, view of motif 2a, 7, and thumb residues. RT, reverse transcriptase.

Side-chain substitutions that reduce activity have at least two roles (Fig. 3, *A*–*C*; see color-coded annotation of side-chain roles in Fig. 1*C*). First, motif 1 K452 and motif 2 R463 are predicted to form salt bridges to the incoming nucleotide triphosphate (Fig. 3*B*) as part of a dNTP-binding pocket that has structurally analogous side chains in retroviral RTs (40, 41, 42). For BoMoC, the K452A substitution results in less severe activity and NTA defects than the R463A substitution, and the K452A enzyme remains capable of template jumping to the annealed primer-template oligonucleotides present in excess (Fig. 2*B*, products labeled Template Jumps). Second, motif 2 I465 and R473 side chains are predicted to make template-strand contacts. The I465 side chain is predicted to stack with the templating base in the active site to promote pairing with the incoming dNTP (Fig. 3*B*), which could explain why BoMoC I465A has reduced NTA and reduced activity (Fig. 2*B*). The R473 side chain is predicted to make polar contacts to the phosphate backbone near the templating base (Fig. 3, *B* and *C*); the absence of this side chain is strongly inhibitory for BoMoC activity (Fig. 2*B*).

BoMoC F534G, with a side-chain substitution in motif 3 that contributes to the dNTP-binding pocket (Fig. 3*B*), showed some loss of activity and the most severe NTA deficiency. A large fraction of cDNA products lacked any 3′ overhang (Fig. 2*B*, +0 products). The closest substrate feature near the F534 side chain is the deoxyribose sugar of the incoming dNTP (Fig. 3*B*). The analogous residue in the RT from Moloney Murine Leukemia Virus, F155, serves as a steric gate against ribonucleotides (the “sugar gate”). Moloney Murine Leukemia Virus RT substitution F155V increased binding and incorporation of NTPs (43). For comparison to BoMoC F534G and Moloney Murine Leukemia Virus RT F155V, we made an analogous amino acid substitution in the group II intron RT from *Eubacterium rectale* (14), purified with the same fusion-tag configuration as BoMoC (29). *Eubacterium rectale* intron RT (termed EuRe) has less NTA activity than BoMoC, but the EuRe sugar gate substitution F146V nonetheless decreased NTA (Fig. 2*B*, compare EuRe to EuRe SG). In addition, a recently published mutagenesis study demonstrated that a similar F143A substitution in a thermostable group II intron RT also decreased NTA (38), which further highlights the importance of this residue in nontemplated nt addition across a broad spectrum of RTs.

The BoMoC motif 2a substitution D500A and palm-domain substitutions L704A and F708A also compromised NTA (Fig. 2*B*), despite side-chain positions outside the dNTP-binding pocket (Fig. 3*D*). In comparison, substitution of the thumb-domain side chains of F753 or I770 (Fig. 3*D*) appeared to slightly increase NTA (Fig. 2*B*). To investigate how these side-chain substitutions influence NTA, we combined the D500A substitution and the F753A substitution to determine whether the NTA defect of D500A BoMoC could be ameliorated by the F753A-substitution activity enhancement. The double-mutant enzyme had cDNA synthesis activity intermediate between BoMoC D500A and BoMoC F753A, but it retained the BoMoC D500A NTA defect (Fig. 2*B*). This finding further resolves cDNA synthesis and NTA as separable activities.

**Figure 4:**
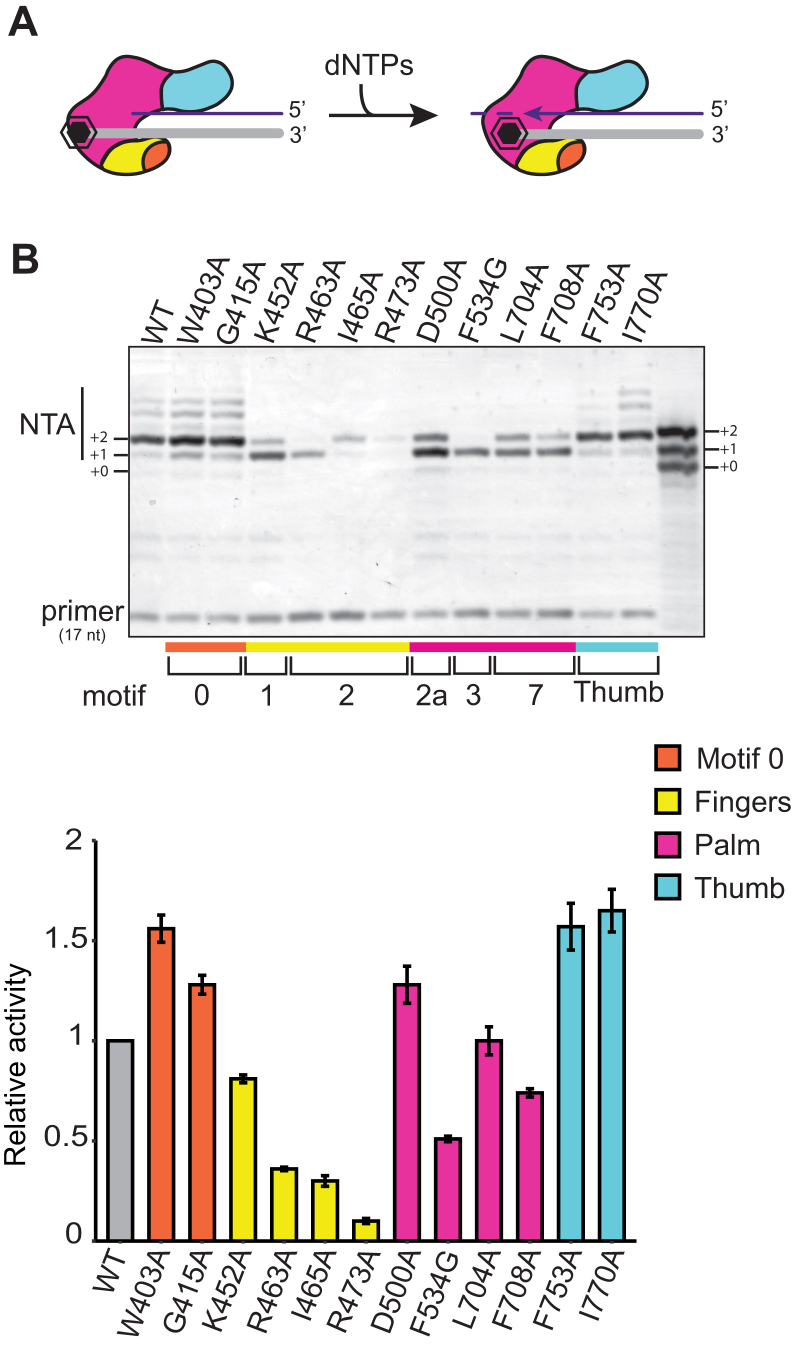
**Independence of NTA from template jumping.***A*, diagram of BoMoC engaged with primer-template duplex with a template 5′ Cy3 group added to inhibit template jumping. A *black hexagon* represents the Cy3 group. *B*, *top*, SYBR-gold stained denaturing urea-PAGE gel of cDNA products of the BoMoC variants. Far right lane has +0, +1, +2 markers for addition of the corresponding number of nt by NTA. *Bottom*, quantification of +0 and NTA cDNA products normalized to WT. cDNA, complementary DNA; NTA, nontemplated nucleotide addition.

To examine the possibility that NTA defects arise from abortive template jumping, analogous to the abortive template copying responsible for “NTA” by T7 RNA polymerase (44), we assayed cDNA synthesis using a primer-template duplex with a template-strand 5′ Cy3 dye (Fig. 4*A*; oligonucleotide sequences are listed in Table 1). This bulky modification disfavors binding of the incoming template necessary for template jumping (29) and would therefore diminish apparent NTA resulting instead from nonprocessive cDNA synthesis. BoMoC variants elongated the recessed primer 3′ end to the template 5′ end with relative activity levels like those observed for fill-in synthesis on the unmodified template (compare Fig. 4*B* to Fig. 2*B*). The enzymes’ relative differences in NTA were also similar comparing across templates, although the product profile with the 5′ Cy3 template shifted to predominantly +2 or +1 nt of NTA (Fig. 4*B*). BoMoC variants with R463A or F534G substitution were the most compromised for NTA under any assay condition. In summary, all the findings described above suggest that NTA activity is particularly sensitive to changes in structural features of the dNTP-binding pocket, and that NTA has separable structural requirements from fill-in cDNA synthesis.

### Separable requirements for NTA and manganese-induced terminal transferase activity

*In vitro*, BoMoC has a second mode of nontemplated primer elongation, which is detected in reactions containing an above-physiological concentration of manganese and therefore of uncertain biological significance. Instead of the NTA extension of the 3′ end of a duplexed cDNA product, the manganese-induced terminal transferase or “tailing” activity extends single-stranded nucleic acids (29). This requires the RT to stably position single-stranded rather than duplexed primer substrate in the active site. Tailing activity differs from NTA in that tailing can add tens or hundreds of nucleotides in a processive manner and can extend ssRNA, enabling new laboratory applications of BoMoC RT (29).

**Figure 5:**
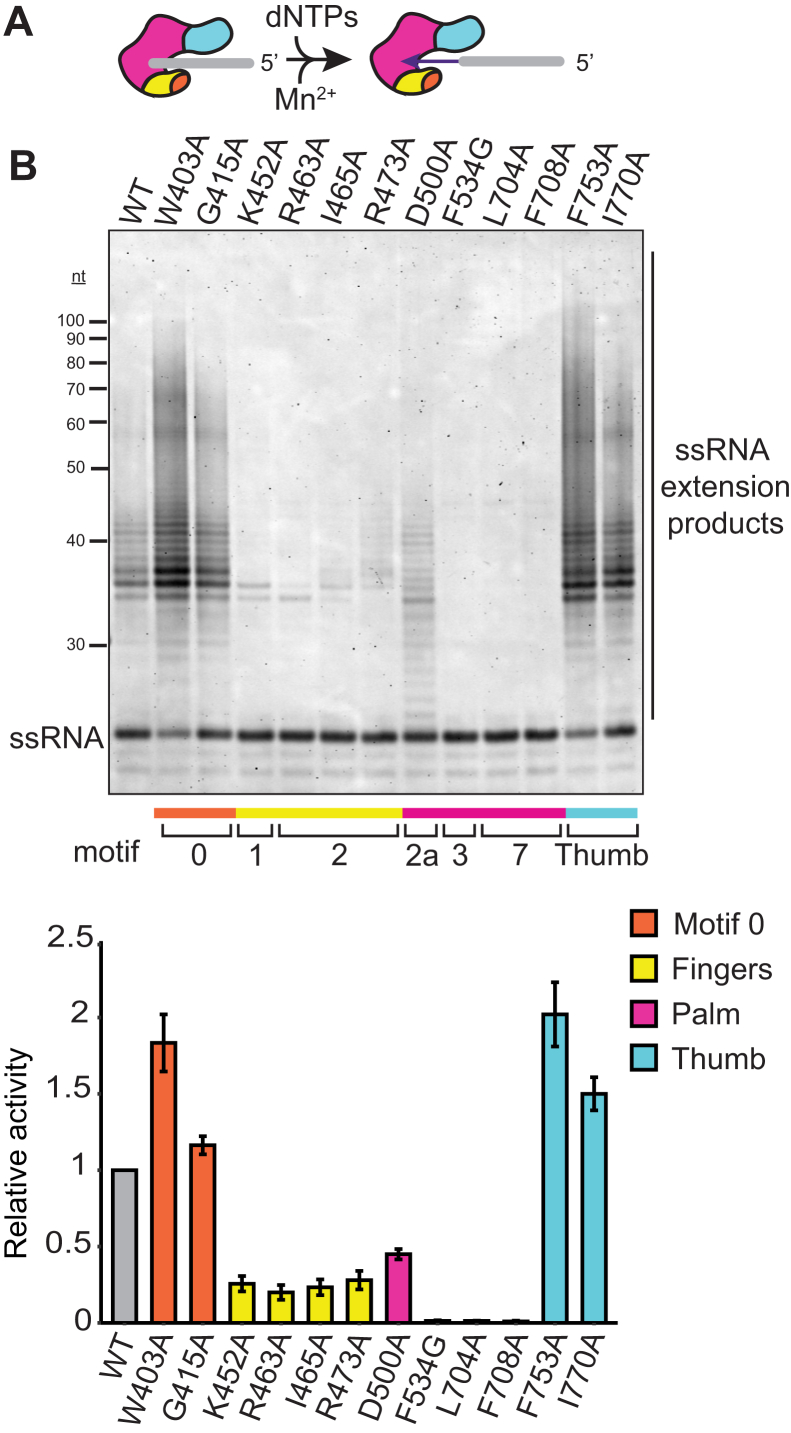
**Tailing activity of BoMoC variants.***A*, diagram depicting BoMoC extension of a ssRNA 3′ end in the presence of manganese (Mn^2+^). *B*, *top*, SYBR-gold stained denaturing urea-PAGE gel of ssRNA extension products. *Bottom*, quantification of ssRNA extension products normalized to WT.

To investigate BoMoC structural requirements for ssRNA tailing in relation to templated cDNA synthesis and NTA, we assayed the BoMoC variants under tailing reaction conditions (Fig. 5*A*; ssRNA oligonucleotide sequence is listed in Table 1). The impact of amino acid substitutions on tailing activity was not directly correlated to impact on cDNA synthesis activity (compare Fig. 5*B* to Fig. 2*B*). Fewer of the BoMoC variants supported near-WT tailing activity than near-WT cDNA synthesis. The sharpest dichotomy was observed for the motif 7 L704A and F708A BoMoC variants: despite near-WT cDNA synthesis activity and some NTA to the cDNA duplex (Fig. 2*B*), these BoMoC variants had undetectable tailing activity (Fig. 5*B*). The motif 7 side chains L704 and F708 are in the “primer grip” motif (45) located near the DNA 3′ end (Fig. 3*D*).

On the other hand, the BoMoC variants with increased cDNA synthesis activity showed increased ssRNA tailing activity, notable for both the motif 0 and thumb-domain substitution variants (Fig. 5*B*). Thumb-domain F753 extends into the duplex minor groove and contacts newly synthesized DNA whereas I770 side chain is predicted to face into a hydrophobic pocket that stabilizes three antiparallel alpha helices of the thumb domain (Fig. 3*D*). Thumb-domain substitutions that reduce the interaction of BoMoC with its template-product duplex could increase activity by increasing enzyme turnover, but how they could cause an increase in ssRNA tailing is less obvious. Increased ssRNA tailing was also observed for the motif 0 side chain substitutions (Fig. 5*B*). Compared to the thumb domain, motif 0 function is not well characterized. BoMoC side chains W403 and G415 flank a loop predicted to contact unpaired template (Figs. 3*C* and S2*A*), on the opposite side of the active site cleft from the thumb domain (Fig. 3*A*).

### Bypass of an NTA requirement for template jumping

**Figure 6:**
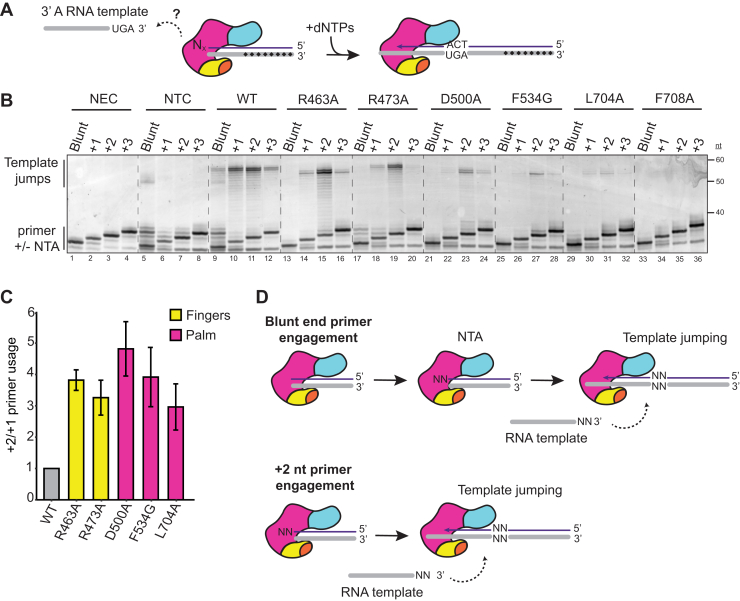
**Rescue of template jumping by a 2 nt primer 3′ overhang.***A*, diagram of +1, +2, or +3 nt primer overhang (represented by N_x_) duplex. *Dashed arrow* indicates potential jump to a 3′A RNA template. *Black diamonds* within RNA template strand (*gray*) indicate 2′ O-methyl modifications of the template 3′ end for increased T_m_. *B*, SYBR-gold stained denaturing urea-PAGE gels of BoMoC-variant cDNA products using blunt, +1, +2, or +3 primer overhangs. No template controls (NTC) lack the 3′A jump template oligonucleotide but have WT BoMoC enzyme added. The NTC reaction with WT BoMoC and blunt primer duplex had a low level of spurious product formation. *C*, quantification of the ratio of template jump products from a +2 nt overhang primer to all jump products from +1 nt overhang primer, normalized to WT enzyme. *D*, *top diagram* depicts BoMoC extension of a blunt-end primer-template duplex by + 2 NTA for successful template jumping. *Bottom diagram* depicts the rescue of template jumping by NTA-deficient BoMoC variants using +2 nt overhang primer-template duplex to bypass the NTA requirement. cDNA, complementary DNA; NTA, nontemplated nucleotide addition.

The template-jumping activity of RTs is useful for laboratory applications such as cDNA library synthesis (3, 29). However, it can also complicate cDNA library production by producing nonnative, chimeric cDNAs (37). Therefore, we sought to understand why most low-NTA BoMoC variants (BoMoC R463A, R473A, D500A, F534G, L704A, and F708A) did not generate template-jumping products (Fig. 2*B*). BoMoC K452A is the exception: it has reduced NTA but still supports template jumping. To this end, we tested template jumping from blunt duplexes or duplexes with primer-strand 3′ overhangs. We assayed +1, +2, and +3 nt 3′ overhangs representative of different lengths of NTA (Fig. 6*A*; oligonucleotide sequences are listed in Table 1). A no enzyme control and no template control were compared to visualize products from NTA without template jumping (Fig. 6*B*, compare lanes 1–4 and 5–8 in the region labeled primer ± NTA). The no template control reaction with WT BoMoC and blunt primer duplex had a low level of spurious product formation, which was reduced when reactions also contained a template RNA oligonucleotide (Fig. 6*B*, compare lanes 5 and 9).

Under assay conditions that are optimal for template jumping, in reactions containing an added oligonucleotide template, WT BoMoC generated detectable cDNA synthesis products with all four primer duplexes, but maximal template jumping occurred with +1 or at most +2 nt of 3′ overhang complementary to the template 3′ end (Fig. 6*B*, lanes 9–12, products labeled Template Jumps). With WT BoMoC, NTA to the input primer-template duplex was maximal on the blunt-end substrate (Fig. 6*B*, lanes 5 and 9; note the products slightly longer than input primer). In comparison, the other BoMoC enzymes showed little if any NTA to the primer duplexes and showed little if any template jumping from the blunt-end or +1 nt overhang duplex (Fig. 6*B*, lanes 13–14, 17–18, 21–22, 25–26, 29–30, 33–34).

To our surprise, most of the low-NTA enzymes that lacked template jumping after cDNA synthesis (Fig. 2*B*) regained the ability to perform cDNA synthesis by template jumping when assayed with the +2 nt 3′ overhang primer duplex (Fig. 6*B*, lanes 15, 19, 23, 27, 31, 35). For quantitative comparison to WT, primer preference was measured as a ratio of primer +2 and +1 usage (Fig. 6*C*; note that F708A BoMoC template jumping could not be quantified against background). The exceptions to primer +2 nt 3′ overhang rescue of template jumping were the BoMoC primer-grip variants with L704A or F708A substitution, which had the weakest rescue of template-jumping activity (Fig. 6*B*, lanes 29–36) despite their WT level of cDNA synthesis fill-in of a recessed primer overhang (Fig. 2*B*). The L704A or F708A side-chains are close to the DNA 3′ end (Figs. 3*D* and S2*B*) and could therefore play a large role in enzyme grip on products with a 3′ overhang. On the other hand, R463A and R473A BoMoC had relatively strong rescue of template-jumping activity using the +2 nt 3′ overhang primer duplex (Fig. 6*B*, lanes 15 and 19), despite having a low activity level for fill-in cDNA synthesis (Figs. 2*B* and 4*B*). R463 and R473 surround the nucleotide-binding pocket and unpaired template (Fig. 3*B*). As would be predicted from its minimal compromise of NTA, I465A BoMoC retained template-jumping activity similar to WT BoMoC (Table 2). We conclude that although NTA does not depend on BoMoC ability to template jump, BoMoC template jumping does depend on NTA or the bypass of NTA using a primer duplex with a primer-strand 3′ overhang.

**Table 2:** Summary of results

Enzyme			Fill-in cDNA synthesis	NTA profile	Tailing	Jumping after cDNA synthesis	Jump from +2/+1 primer duplex
		WT		3–4	++	+	1
Motif		W403A	↑	3–4	++	+	n.d.
		G415A	↑	3–4	++	+	n.d.
Fingers		K452A	↓	0–3	+	+	∼1
		R463A	↓	0–2	+	–	>3
		I465A	↓	2–3	+	–	∼1
		R473A	↓	3	+	–	∼3
Palm		D500A	↑	0–3	+	–	>3
		F534G	↓	0	–	–	>3
		L704A	↑	0–3	–	–	∼3
		F708A	↑	0–3	–	–	n.d.
Thumb		F753A	↑	3–4	++	+	n.d.
		I770A	↑	3–4	++	+	n.d.

Up-arrows indicate an increase in activity in fill-in cDNA synthesis assays compared to WT and down-arrows indicate a decrease in activity. NTA profile range is given as the most predominant NTA products evident in Figure 2*B*. Tailing activity is categorized into three levels: two plus signs indicate WT or stronger signal, one plus sign indicates weak activity, and a minus sign indicates no activity. Template jumping after initial primed cDNA synthesis is similarly demarcated by plus and minus signs. “Jump from +2/+1 primer duplex” gives the ratio of template-jumping product synthesis using primer duplexes with +2 or +1 nt overhang, with representative data shown in Figure 6. Enzymes that were not tested or results that were not reliably quantified because of low signal are marked n.d. for not determined. Results in the “Jump from +2/+1 primer duplex” column for BoMoC K452A and I465A are not shown but were performed parallel to assays shown in Figure 6; the primer preference for template jumping by BoMoC K452A and I465A was similar to that of WT BoMoC.

## Discussion

### Structure/function relationship for a non-LTR RT

*B. mori* R2 protein is a favorable system for characterization of the biochemical activities of a non-LTR retroelement RT. Previous mutagenesis studies using the full-length R2 protein explored the sequence requirements for nucleic acid binding and EN activity, targeting regions of the protein N- and C-terminal to the core RT domain (46, 47, 48, 49). The BoMoC variants described in this work specifically tease apart RT active-site structural requirements for cDNA synthesis, NTA, and template jumping (Table 2), enabling future studies of how these RT activities support non-LTR retroelement mobility. In addition, our results provide a basis for comparison of the active-site architecture of a non-LTR RT and the active sites of retroviral and intron RTs and viral RdRPs. Our findings indicate that protein side chains of the nucleotide-binding pocket and side chains involved in unpaired-template interaction immediately adjacent to the primer duplex have critical contributions to both cDNA synthesis activity and NTA, whereas in contrast motif 0 and thumb-domain side chains do not. The particular motif 0 and thumb-domain substitutions examined in this work actually improve *in vitro* enzyme function in cDNA synthesis and as a terminal transferase in manganese-dependent ssRNA tailing.

The mechanism by which BoMoC thumb-domain F753A and I770A substitutions increase cDNA synthesis activity is likely to involve reduced stability of enzyme interaction with the product-duplex minor groove. A thermostable group II intron RT has thumb domain Y325 (15) in similar position to BoMoC F753. Having a bulky hydrophobic side chain monitoring minor groove geometry serves as a fidelity check and increases affinity for product DNA (45). Thumb-domain contact with the minor groove contributes to the particularly high processivity of the EuRe group II intron RT (14). Likewise, R2 RT thumb-domain side chains F753 and I770 could be important for the high processivity essential to full-length copying of non-LTR retroelement template RNAs, but assays used in this work do not demand high processivity. When performing cDNA synthesis on relatively short RNA templates, reduced interaction of BoMoC with product duplex could favorably increase the rate of enzyme release and thus allow more cDNA synthesis overall.

In previous work, motif 0 substitutions in full-length *B. mori* R2 RT or a group II intron RT decreased enzyme activity and inhibited template jumping (15, 47). Those studies targeted residues in a conserved P-G-hydrophobic-D-G motif (Fig. S2*A*, BoMoC sequence PGPDG). When we made BoMoC single amino acid substitutions of each of the previously targeted residues, we observed a similar phenotype (data not shown). The BoMoC motif 0 substitutions assayed in this work were instead made at the base of what is known as Motif G or the “G Loop” of RdRPs (50) or the “RT 0 lid” of a group II intron RT that contains the conserved P-G-hydrophobic-D-G sequence (15). Here, the motif 0 substitutions, W403A and G415A, surprisingly increased BoMoC cDNA synthesis and tailing activities without an apparent change in NTA. Of particular note, removal of the large hydrophobic side chain of W403 did not inhibit template jumping. We suggest that our motif 0 substitutions may have directly or indirectly, through changes in positioning of other motif 0 amino acids, reduced constraints on unpaired template entry into the active site. The WT sequence of motif 0 could be more important for BoMoC cDNA synthesis on its highly structured cellular template than on the oligonucleotide templates used in this work. We are developing cellular assays of RT-mediated gene insertion that will enable future testing of the consequences of the BoMoC motif 0 side-chain substitutions on retroelement mobility.

### Template-jumping requirements of a non-LTR RT

Template jumping is a shared property of retroviral, group II intron, and non-LTR retroelement RTs assayed *in vitro* (3). What governs the ability of a polymerase to support template jumping has been challenging to conclude, largely because of uncertainties in interpreting the comparison of results obtained in different laboratories with different enzymes, templates, and reaction conditions. For example, full-length *B. mori* R2 RT was not thought to support template jumping from a duplexed primer (22), whereas BoMoC does (29). This disparity is more likely to reflect differences in purification and assay conditions than in inherent enzyme properties.

Against our initial expectation, some BoMoC variants compromised for template jumping could be rescued for this activity using a primer duplex with a 2 nt 3′ overhang. This finding bolsters a conclusion that template jumping requires prior NTA to a cDNA or primer 3′ end (Fig. 6*D*). We also conclude that dNTP-binding pocket integrity is not critical for template jumping, if the NTA requirement is bypassed by primer design. However, even with bypass of the NTA requirement, BoMoC L704A and F708A variants remained severely compromised for template jumping. Relatively weak rescue of template jumping for these enzymes correlates with their sharply decreased ssRNA tailing activity. How template jumping and tailing are dependent on side chains within the primer grip motif (Fig. S2*B*), and other side chains near the primer 3′ end, will be interesting to investigate in more detail. Independent of the mechanism, the use of BoMoC L704A and F708A enzymes with robust cDNA synthesis activity but severely inhibited template jumping can enable new RT laboratory applications.

For *B. mori* R2 RT and BoMoC, the probability that a 3′ overhang created by NTA matches an incoming template 3′ end strongly depends on reaction conditions (23, 29). In reactions with balanced concentration of the four dNTPs, the RT adds predominantly non-templated adenosines to generate an at least 3 nt NTA overhang, which is nonproductive for template jumping (29). We suggest that for non-LTR retroelement RTs, as for group II intron and retroviral RTs, template jumping may not be physiologically necessary for retroelement/retrovirus proliferation. Indeed, the inserted cDNA chimeras characterized in human LINE-1 mobility assays arise from preligation of the retroelement transcript to a noncoding RNA, rather than copying of discontinuous templates (51). In future studies, it will be of interest to dissect the biological requirements for template jumping in retroelement mobility using the structure/function insights from this work.

## Experimental procedures

### Construction of expression vectors and protein purification

The 2Bc-T vector encoding N-terminal maltose-binding protein and C-terminal six-histidine tagged BoMoC (29) was used as a template for site-directed mutagenesis. Intended changes were confirmed by DNA sequencing. Plasmids were transformed into BL21(DE3) cells and plated, then a single colony was grown in 2xYT media and induced at A_600_ 0.6 to 0.8 at 16 °C overnight with 1 mM Isopropyl β-d-1-thiogalactopyranoside. Cells were harvested by centrifugation at 4000 rpm and lysed in 20 mM Tris pH 7.5, 1 M NaCl, 1 mM MgCl_2_, 0.2% NP-40, 10% glycerol, 1 mM DTT, and protease inhibitors. Two-step purification was initiated by allowing lysate protein to bind Nickel-NTA resin for 2 h at 4 °C. After binding, resin was washed three times with 20 mM Tris pH 7.5, 1 M KCl, 20 mM imidazole, 10% glycerol, 0.1% NP-40, and 1 mM DTT. Proteins were subsequently eluted in 20 mM Tris pH 7.5, 0.8 M KCl, 10% glycerol, 1 mM DTT, and 500 mM imidazole. Eluted protein was size-fractionated on a HiLoad 16/600 Superdex 200 pg column in the Nickel-NTA elution buffer without imidazole. Pooled fractions were concentrated using Amicon centrifugal filter units, and protein concentration was quantified by bicinchoninic acid assay. BoMoC protein integrity and purity were validated by Coomassie blue staining after sodium dodecyl sulfate - polyacrylamide gel electrophoresis. Enzyme aliquots were stored at −80 °C. Enzyme working stocks were diluted to 10 μM protein in 25 mM Tris pH 7.5, 200 mM KCl, 400 mM (NH_4_)_2_SO_4_, 50% glycerol, and 2 mM DTT, and stored at −20 °C. Bacterial RT proteins were purified as previously described (29).

### Protein structure prediction

The Phyre2 Protein Fold Recognition server was used for protein structure prediction (52). Motifs -1 through the thumb were submitted, of which 85% (435 residues) were modeled at >90% accuracy across two templates described in Results. Intensive mode with default parameters was used. Residues modeled *ab initio* were omitted from the 3D model represented in this work (see Fig. 1 legend). Figure panels containing structure predictions were made using ChimeraX (53).

### Activity assays

For cDNA synthesis and NTA assays, 20 μl reactions with 0.5 μM RT protein were carried out in RT buffer (20 mM Tris pH 7.5, 150 mM KCl, 5 mM MgCl_2_, 1 mM DTT, 2% PEG-6000, and 500 μM dNTPs), and 200 nM primer duplex. Reactions proceeded for 10 min at 37 °C, followed by heat inactivation at 65 °C for 5 min, then addition of 0.5 μg/μl final concentration of RNase A (Sigma, R6513) with incubation for 15 min at 50 °C. The reactions were stopped with 50 mM Tris pH 7.5, 20 mM EDTA, and 2% SDS. Products were extracted with phenol: chloroform: isoamyl alcohol (25:24:1), isopropanol precipitated using 10 μg glycogen as carrier, and air-dried for 5 min before resuspension in 5 μl 2× formamide loading dye (95% deionized formamide, 0.025% bromophenol blue (w/v), 0.025% xylene cyanol (w/v), and 5 mM EDTA). Products were separated on 15% denaturing urea-PAGE gels then stained using SYBR Gold and imaged by Typhoon Trio.

For terminal transferase tailing assays, the reactions proceeded as above except 5 mM MgCl_2_ and 200 nM primer-duplex were substituted with 5 mM MnCl_2_ and 200 nM ssRNA. Products were processed for denaturing PAGE as described above but without the RNase step.

For assays testing rescue of template jumping, nucleotide concentrations were adjusted to 200 μM dTTP, 40 μM dCTP, 40 μM dGTP, and 20 μM dATP. Universal primer duplexes were added at 50 nM, and 3′A jump-acceptor template was added at 200 nM. The reactions were carried out at 37 °C for 10 min, followed by analysis as described above.

### Quantification and statistical analyses

All gel quantification was done with ImageJ. All graphical data represents mean ± standard error of three technical replicates wherein three separate reactions were performed and reaction products analyzed on different gels.

## Data availability

All data are contained within the article.

## Supporting information

This article contains supporting information (54).

## Conflict of interest

BoMoC variants with improved properties are included in patent applications filed by University of California, Berkeley, with S. C. P., H. E. U. and K. C. as named inventors. H. E. U. and K. C. are the founders of Karnateq Inc., which licensed the RT technology.
