# Supplementary material for: Separable structural requirements for cDNA synthesis, nontemplated extension, and template jumping by a non-LTR retroelement reverse transcriptase
Source: J Biol Chem. 2022 Jan 21;298(3):101624. doi: 10.1016/j.jbc.2022.101624 (PMC8857657; doi:10.1016/j.jbc.2022.101624)
Supplement: Supplemental Figures S1 and S2 [file mmc1.pdf]

Separable structural requirements for cDNA synthesis, non-templated extension, and template jumping by a non-LTR retroelement reverse transcriptase

Sydney C. Pimentel, Heather E. Upton, Kathleen Collins

Department of Molecular and Cell Biology

University of California at Berkeley

Berkeley, CA 94720-3202

Supporting Information Figure legends

Figure S1. Sequence conservation and RT motif boundaries predicted for BoMoC. Amino acids 275-798 of *B. mori* R2 protein were submitted to the ConSurf server (Berezin C., Glaser F., Rosenberg J., Paz I., Pupko T., Fariselli P., Casadio R. and Ben-Tal N. (2004) ConSeq: The Identification of Functionally and Structurally Important Residues in Protein Sequences. *Bioinformatics,* 20, 1322-1324) using default parameters: multiple sequence alignment generated using MAFFT (Multiple Alignment using Fast Fourier Transform) and homologues collected from UNIREF90. Motif amino acid boundaries are notated above sequence with residue numbering from full-length *B. mori* R2 protein. Asterisks indicate residues probed for function by mutagenesis in this study.

Figure S2. Structure modeling for motif 0 and palm side chains. (A) The circled PGPDG sequence of BoMoC motif 0 was not examined in this work. (B) View of L704 and F708 showing predicted proximity to DNA primer 3’ end.

Figure S1.

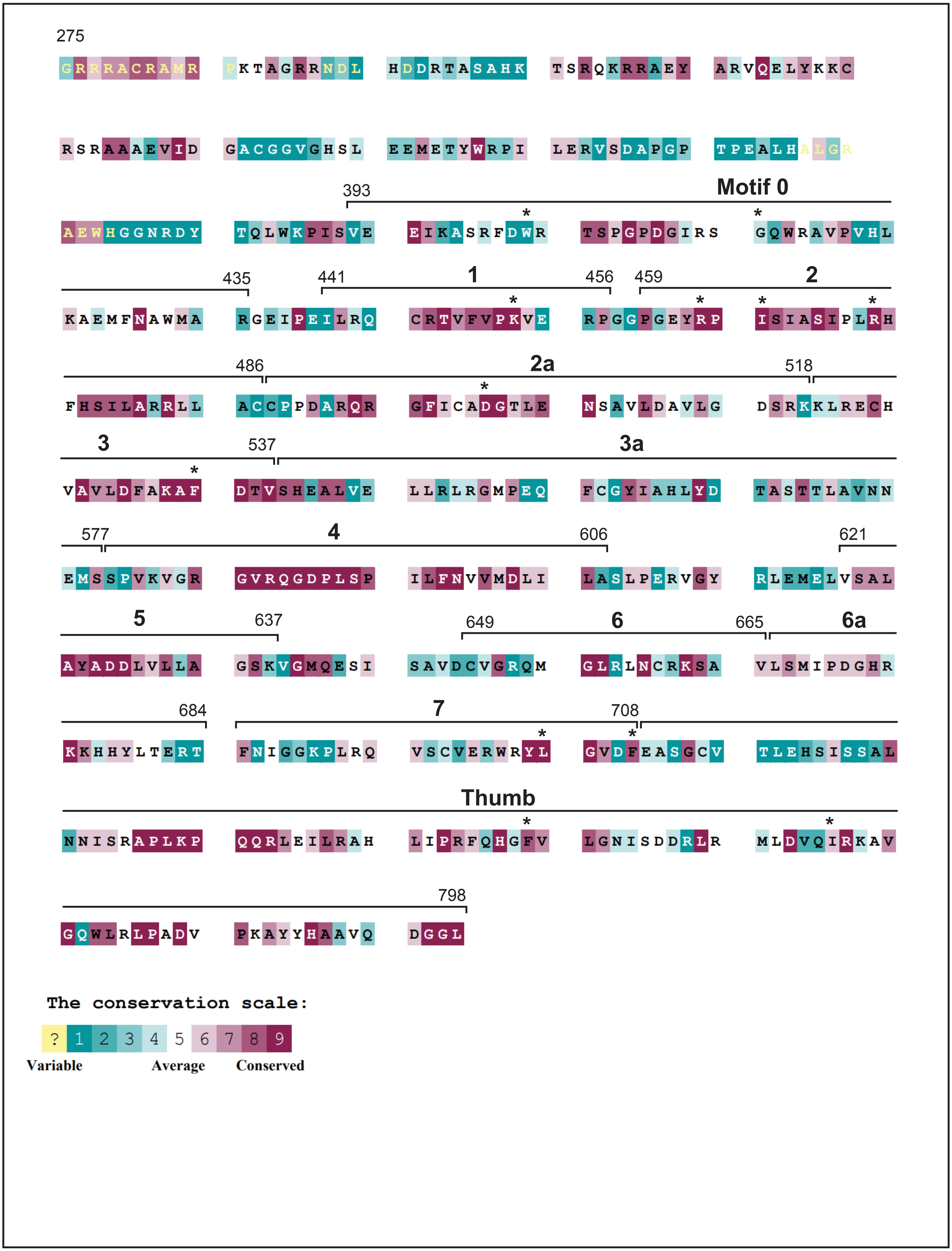
275
GRRRACRAMR PKTAGRRNDL HDDRTASAHK TSRQKRRAEY ARVQELYKKC
RSRAAAEVID GACGGVGHSL EEMETYWRPI LERVSDAPGP TPEALHALGR
393 Motif 0
AEWHGGNRDY TQLWKPISVE EIKASRFDWR TSPGPDGIRS GQWRAVPVHL
435 441 1 456 459 2
KAEMFNAWMA RGEIPEILRQ CRTVFVPKVE RPGGPGEYRP ISIASIPLRH
486 2a 518
FHSILARRLL ACCPPDARQR GFICADGTLE NSAVLDAVLG DSRKKLRECH
3 537 3a
VAVLDFAKAF DTVSHEALVE LLRLRGMPEQ FCGYIAHLYD TASTTLAVNN
577 4 606 621
EMSSPVKVGR GVRQGDPLSP ILFNVVMDLI LASLPERVGY RLEMELVSAL
5 637 649 6 665 6a
AYADDLVLLA GSKVGMQESI SAVDCVGRQM GLRLNCRKSA VLSMIPDGHR
684 7 708
KKHHYLTERT FNIGGKPLRQ VSCVERWRYL GVDFEASGCV TLEHSISSAL
Thumb
NNISRAPLKP QQRLEILRAH LIPRFQHGFV LGNISDDRLR MLDVQIRKAV
798
GQWLRLPADV PKAYYHAAVQ DGGL
The conservation scale:
? 1 2 3 4 5 6 7 8 9
Variable Average Conserved

Figure S2.

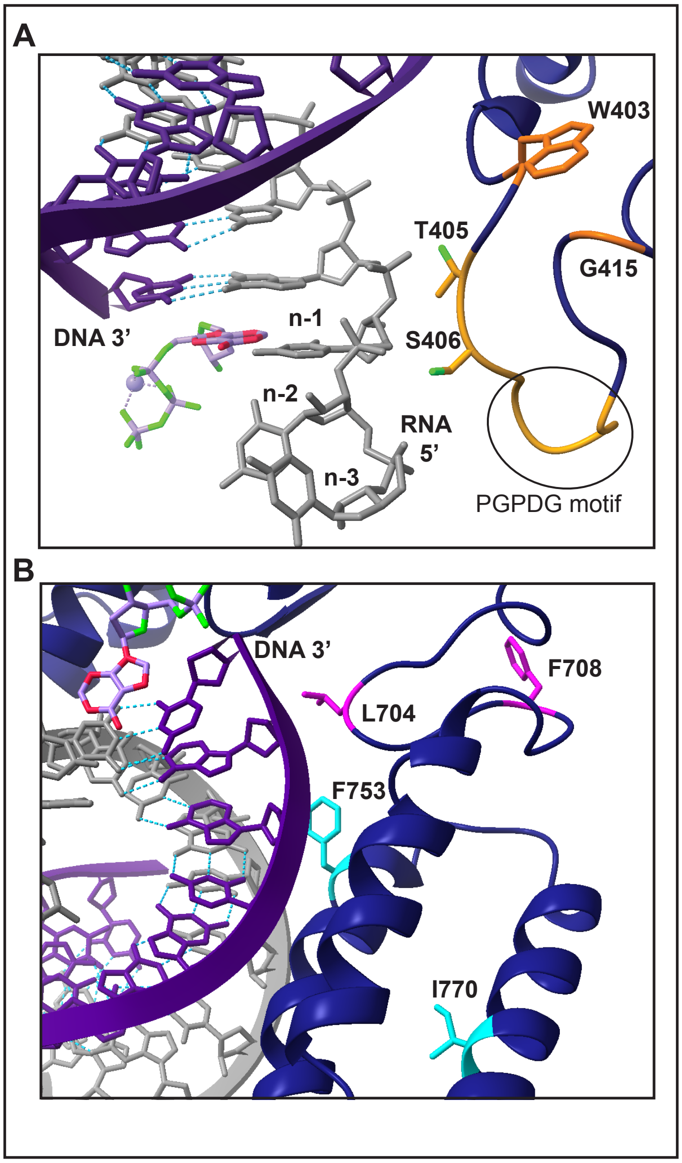
A
W403
T405
G415
n-1
DNA 3'
S406
n-2
RNA
5'
n-3
PGPDG motif
B
DNA 3'
F708
L704
F753
I770
